# Intussusception in preterm neonates: A systematic review of a rare condition

**DOI:** 10.1186/s12887-021-03065-5

**Published:** 2021-12-24

**Authors:** Mostafa Kotb, Mostafa Abdelatty, Hayssam Rashwan, Yasmine AbdelMeguid, Ahmed Elrouby

**Affiliations:** 1 Pediatric Surgery Department, Alexandria Faculty of Medicine, Alexandria, 21615 Egypt; 2 Pediatrics Department, Alexandria Faculty of Medicine, Alexandria, Egypt

**Keywords:** intussusception, preterm, necrotizing enterocolitis

## Abstract

**Background:**

While necrotizing enterocolitis (NEC) is a prevalent condition in preterm neonates admitted to neonatal intensive care unit (NICU), intussusception is exceedingly uncommon and often overlooked. This is due to the fact that they share many clinical characteristics. The initial misdiagnosis of intussusception in preterm neonates (IPN) especially has led to a delay in their management, which increases the risk of developing compromised bowel. Additionally, it is difficult to reach a diagnosis as neonatal intussusception does not have any classical radiological signs even when contrast enema is used. This systematic review is based on the published literature including case reports and case series to review the clinical features of IPN and how to differentiate it from NEC in order to shed the light on this rare disease and how having a high index of suspicion would help practitioners to make an early and accurate diagnosis

**Methods:**

A systematic literature search to report all cases of relevant articles that reported IPN till date. All cases that were born before 37 weeks gestational age, presented within the neonatal period and having well established documentation were included in the study. Any case that did not have these criteria was excluded from our study.

**Results:**

Only 52 cases met these criteria during the period from 1963 till date. An average of 10 days had elapsed before the cases were confirmed to have IPN either clinically or intraoperatively. The most frequent manifestations were abdominal distension and bilious gastric residuals, occurring in 85% and 77% of the cases respectively, followed by bloody stools in 43% of cases. However, this triad was present only in approximately one-third of the cases. Only 13 cases were diagnosed as having intussusception preoperatively. About two thirds of the intussusception were located in the ileum. Pathological lead point was present in 7 cases only; 4 of them were due to Meckel’s diverticulum. Nine cases only out of the 52 cases with IPN died.

**Conclusion:**

It is crucial to detect the clues for diagnosis of intussusception because in contrast to NEC, it is unresponsive to conservative management, affects the viability of the bowel and surgery is essential.

## Background

Although intussusception is the commonest etiology of bowel obstruction in infants, it is uncommonly encountered in neonates. Moreover, it is extremely uncommon in preterms [1]. Clinically, it could be commonly confused with the more common disorder in neonates, necrotizing enterocolitis (NEC), as both share common symptomatology. This includes abdominal distension, bilious emesis, bloody stools and feeding difficulties [2].

The initial misdiagnosis of intussusception in neonates especially preterms has led to a delay in their management, which increases the risk of developing compromised bowel. In addition, it is difficult to reach a diagnosis as neonatal intussusception does not reveal any classical radiological signs even when contrast enema is used. Moreover, contrast enemas may even be hazardous as it may increase the risk of bowel perforation as in most cases the bowel is already compromised at the time of investigation. However, in order to reach a successful management of intussusception in preterm neonates (IPN), a timely and accurate diagnosis is required [3].

Therefore, we conducted the current study based on the systematic review of the published literature including case reports and case series to review the clinical features of IPN and how to differentiate it from NEC in order to shed the light on this rare disease and how having a high index of suspicion would help practitioners to make an early and accurate diagnosis. Although case reports are usually not an integral component of systematic reviews, in rare diseases they should be included to attain a comprehensive overview of the state of research and provide useful information especially when other types of studies are not available.

## Methods

An extensive systematic literature search was done to identify relevant articles that reported intussusception in preterm neonates (IPN). MEDLINE/PubMed, Google Scholar and Science direct databases were searched using key words: “preterm, newborn(s), and intussusception” with no restriction on publication language and date. Literature review was completed on 4 February 2021. To determine their relevance, the title and abstract of all potentially relevant papers were read. Full articles were also scrutinized should the title and abstract were unclear. All cases included in the analysis met the following criteria:

- Gestational age <37 weeks,
- Onset of symptoms within the neonatal period,
- Well established documentation as regards patients’ demography, clinical presentation and intraoperative confirmation of intussusception.

We excluded reports discussing intussusception in full-term neonates, antenatal intussusception and those who are lacking clinically relevant data. Moreover, reviews of previously published papers were excluded to prevent case duplication. The majority of articles were individual case reports, whilst the remaining were case series. All the studies obtained from the search were sent to EndNote X9 software to sum up all studies and track potential duplications and remove these duplicates.

There are challenges to research on rare and heterogeneous conditions. However, case reports and case series occupy an important role as the preliminary data source for such conditions, and when carefully performed, systematic reviews of case reports and case series can provide a useful addition to evidence-based medicine.

Based on ROBINS-I (“Risk Of Bias In Non-randomized Studies - of Interventions), reports were assessed according to the following domains: bias in confounding, participants’ selection, classification of intervention, deviation from intended intervention, missing data, outcomes’ measurement and selection of the reported result. It was interpreted as low, moderate, serious and critical risk of bias [4]. The quality of evidence of each citation was rated using a conceptual scheme for evaluating the quality of a case report designed by Pierson [5]. This scheme evaluates the validity of a case report based on five components: documentation, uniqueness, educational value, objectivity, and interpretation, yielding a score with a maximum of 10 (> 5 suggests a valid case report) [5].

Next, two reviewers independently extracted data from each study using a standardized form that highlight data regarding patient clinical features, investigations, intraoperative findings, co-morbidities and outcome. A third author managed to resolve any inconsistencies between both reviewers. This systematic review was conducted in accordance with the Preferred Reporting Items for Systematic Reviews and Meta-Analyses (PRISMA). Categorical variables of those patients were expressed as frequencies and numeric ones as mean ± standard deviation, median and range.

## Results

**Fig. 1:**
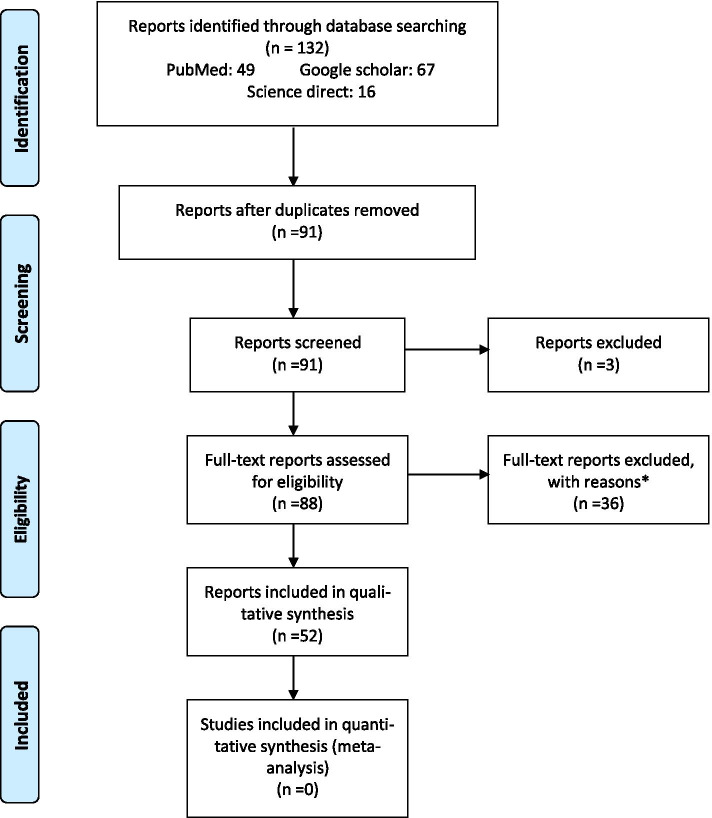
PRISMA IPD Flow Diagram: Flow chart of literature search

The literature search yielded 91 citations related to our topic. After applying inclusion and exclusion criteria, 39 reports were excluded for the following reasons: Three had scanty patients’ data, 3 cases presented after the neonatal period. In addition, 20 were excluded because the cases were full-term, 12 because they were intra-uterine intussusceptions and one was a review article (Figure 1). According to the evaluation criteria cited by Pierson, 44 case reports (85%) scored >5, indicating an overall fair quality of evidence from those cited reports.

Based on the previously mentioned strict criteria, only 52 cases met these criteria during the period from 1963 till date [6–48] (Table 1 and Table 2). Gender was mentioned in all cases. Of these, 38 patients (approximately 73%) were males, whilst 14 (27%) were females, i.e. male-to-female ratio 2.7:1. The mean age of presentation was 10.6 days, while the median was 9.5 days. An average of 9.8 ± 7.5 days for the diagnosis to be confirmed was noted.

**Table 1:** Studies describing the patients of IPN

**Author**	**sex**	**Gest. age**	**Weight**	**Age at onset**	**Age at surgery**	**Abd. distension**	**Gastric residue**	**Bloody stools**	**Abd. Mass**	**Location**	**Perforation**	**Outcome**
Spencer et al [3]	F	28	Not given	Not given	Died preop	Yes	No	No	No	Ileum	Yes	Died
Yoo et al [4]	M	34	2140	2	2	Yes	Yes	No	No	Ileocolic	Yes	Survived
Stine et al [5]	F	28	970	6	33	Yes	Yes	Yes	No	Ileum	No	Survived
Smith et al [6]	F	29	910	21	43	Yes	Yes	Yes	No	Ileum	No	Survived
Glick et al [7]	M	28	1070	14	17	Yes	No	Yes	Yes	Ileum	Yes	Survived
Carman et al [8]	M	25	740	9	15	Yes	Yes	Yes	No	Ileum	No	Survived
Blair et al [9]	M	26	720	8	11	Yes	Yes	No	No	Ileocolic	Yes	Survived
Rausin et al [10]	F	29	1080	5	8	No	Yes	No	No	ileum	Yes	Survived
Farstad et al [11]	M	27	1160	4	14	Yes	No	No	No	Ileum	No	Survived
	M	24	720	14	28	Yes	Yes	No	No	Ileum	Yes	Died
Price et al [12]	M	26	765	15	54	Yes	Yes	Yes	Yes	Jejunum Jejunum	Yes	Survived
	M	30	1240	4	6	Yes	Yes	Yes	No		Yes	Survived
**Author**	**sex**	**Gest.** **age**	**Weight**	**Age at onset**	**Age at surgery**	**Abd. distension**	**Gastric residue**	**Bloody stools**	**Abd. Mass**	**Location**	**Perforation**	**Outcome**
Iutchtman et al [13]	F	26	970	15	20	Yes	No	Yes	Yes	Ileum	No	Survived
	M	28	820	16	18	Yes	No	Yes	No	Jejunum	No	Survived
Puvabanditsin et al [14]	M	26	690	18	35	No	Yes	Yes	No	Jejunum	No	Survived
Mooney et al [15]	M	27	893	11	27	Yes	Yes	No	No	Ileum	No	Survived
Reguerre et al [16]	M	29	1300	15	28	No	Yes	No	No	Ileum	Yes	Survived
Wang et al [17]	M	32	2328	6	21	Yes	Yes	Yes	Yes	Ileum	Yes	Survived
	M	27	950	10	22	Yes	Yes	Yes	No	Ileum	No	Survived
	F	30	1500	14	21	Yes	Yes	Yes	No	Ileum	Yes	Died
Stanely et al [18]	M	32	1205	9	Not given	Yes	Yes	No	No	Ileum	Yes	Survived
Gorgen-Pauly et a l[19]	M	29	995	6	13	Yes	Yes	Yes	Yes	Ileum	Yes	Survived
Hirokawa et al [20]	M	23	630	9	14	Yes	No	No	No	Ileum	Yes	Died
Woo Goo et al [21]	M	27	1185	15	21	Yes	No	No	No	Ileum	Yes	Survived
Margentha et al [22]	M	28	1200	3	10	Yes	Yes	No	No	Jejunum	No	Survived
**Author**	**sex**	**Gest. age**	**Weight**	**Age at onset**	**Age at surgery**	**Abd. distension**	**Gastric residue**	**Bloody stools**	**Abd. Mass**	**Location**	**Perforation**	**Outcome**
Nock et al [23]	M	24	649	9	Not given	Yes	Yes	Yes	No	Ileum	Yes	Survived
Avansino et al [24]	M	24	608	14	34	Yes	No	Yes	No	Jejunum	yes	Survived
	M	30	1130	3	5	Yes	No	No	No	Ileum	Yes	Survive
Biarge et al [25]	F	26	610	30	31	Yes	Yes	No	Yes	Jejunum	No	Survived
Ueki et al [26]	M	34	1940	1	1	No	No	No	No	Ileum	No	Survived
	M	35	2242	1	6	Yes	Yes	No	No	Ileum	No	Survived
Slam et al [27]	M	25	690	18	53	Yes	No	No	No	Multiple	No	Died
Loukas et al [28]	F	34	2120	7	10	Yes	Yes	No	No	Ileum	No	Survived
Boubal et al [29]	F	35	2190	2	4	No	Yes	No	No	Jejunum	No	Survived
Kim et al [30]	M	25	Not given	Not given	39	Yes	Yes	No	No	Multiple	Yes	Survived
Shad et al [31]	M	36	1760	12	12	Yes	Yes	Yes	No	Ileocolic	No	Survived
Shima et al [32]	M	24	588	20	Died preop	No	Yes	Yes	No	Ileum	No	Died
Altuntas et al [33]	F	27	1000	7	12	Yes	Yes	Yes	Yes	Ileum	Yes	Survived
Kim et al [34]	F	23	640	12	20	Yes	Yes	No	No	Multiple	Yes	Survived
Taşkinlar et al [35]	F	25	725	11	12	Yes	Yes	No	No	Ileum	Yes	Survived
	F	29	700	8	8	Yes	Yes	No	No	Ileum	Yes	Survived
	M	31	1800	15	18	Yes	Yes	Yes	No	Ileum	No	Survived
Prakash et al [36]	M	32	1300	11	Not given	Yes	Yes	Yes	No	Ileocolic	No	Died
Park et al [37]	M	26	834	23	80	Yes	Yes	No	No	Multiple	Yes	Died
Patel et al [38]	F	35	2200	21	Not given	Yes	Yes	No	No	Ileum	Yes	Survived
Aydin et al [39]	M	30	2030	17	17	No	Yes	Yes	Yes	Ileum	No	Survived
Tempmalai et al [40]	M	29	1190	4	6	Yes	Yes	No	No	Ileum	Yes	Survived
Diwakar et al [41]	M	25	930	6	14	Yes	No	No	No	Multiple	Yes	Survived
Pawar et al [42]	M	28	1200	8	9	Yes	Yes	No	No	Ileum	Yes	Survived
Raza et al [43]	M	34	2296	2	3	No	Yes	No	No	Colonic	No	Survived
Kotb et al [44]	M	33	1420	21	28	Yes	Yes	Yes	No	Jejunum	No	Survived
Hukeri et a l[45]	M	30	1160	4	6	Yes	Yes	No	No	Jejunum	No	Died

**Table 2:** Summary of the main features and clinical manifestations of IPN cases

	Mean ± SD	Median	Range		
**Gestational age (weeks)**	28.5 ±3.49	28	23-36		
**Birth weight (g)**	1182 ±526	1035	588-2328		
**Age at onset (days)**	10.6 ±6.57	9.5	1-30		
**Age at surgery (days)**	20 ±15.7	17	1-80		
**Delay (days)**	9.8 ±7.5	5	0-57		
**Clinical manifestations**		**Location**		**Treatment**	
Abdominal distention	44 (85%)	Ileoileal	32 (61%)	Anastomosis	24 (46%)
Gastric residue	40 (77%)	Jejunojejunal	10 (19%)	Stoma	18 (34%)
Bloody stool	22 (43%)	Ileocolic	4 (8%)	Reduction	2 (4%)
Abdominal mass	8 (16%)	Colocolic	1 (2%)	Autopsy	2 (4%)
		Multiple	5 (10%)	Pull through	1 (2%)
**Sex (male:female)**	38:14	**Perforation**	27 (53%)	Not given	5 (10%)
**Mortality**	9 (17%)	**Lead point**	7 (14%)		

Abdominal distension and bilious gastric residuals were the most common symptoms, occurring in 85 percent and 75 percent of cases, respectively, followed by bloody stools in 44 percent of cases. This triad, however, was only seen in about one-third of the cases. The existence of an abdominal mass on palpation was uncommon, with only 8 instances displaying this symptom. All of the infants had necrotizing enterocolitis (NEC) prior to surgery, with the exception of 13 who were identified with intussusception prior to surgery, either clinically by feeling the sausage-shaped mass or radiologically by demonstrating the characteristic target sign on ultrasonography. The latter accurately diagnosed intussusception in 11 patients only in a total of 17 patients underwent an ultrasound (US) examination yielding a sensitivity of 65%. The remaining cases did not undergo US examination either due to the presence of pneumo-peritoneum dictating immediate exploration [43], or the strong belief that the diagnosis was NEC at the time of examination where US would not be needed [47].

The most common location of IPN was the small bowel, where ileo-ileal intussusception was present in 32 cases (61%). Next in frequency was jejuno-jejunal in 9 cases, ileo-colic in 4 and colo-colic in two cases only. Multiple intussusceptions were reported in 5 cases only. In 53% of the cases, perforation was evident including two cases having the perforation following contrast enema. While the majority of the cases did not show a primary cause, pathological lead point was present in 8 cases only; 4 of them were due to Meckel’s diverticulum, 2 due to meconium plugs and only a case had postoperative intussusception following sigmoid colostomy for imperforate anus and another one due to abdominal lymphangioma. Nine out of the 52 cases (17%) with IPN died; sepsis was the cause of death in 6 cases, whilst the cause of death was not clearly reported in the remaining 3 cases.

## Discussion

Intussusception is the most common cause of intestinal obstruction in infancy; nonetheless, it is an extremely rare in neonates, particularly among premature infants. It comprises for as low as 3% of neonatal intestinal obstruction and 0.3% of all cases of intussusception [49].

IPN could be easily confused with the highly prevalent disease among neonatal age group, necrotizing enterocolitis (NEC), leading to delay in diagnosis with an average of 9.5 ± 7.3 days in most studies [27]. This delay can lead to higher possibility of intestinal necrosis and subsequent perforation in preterm infants with intussusception [22, 28]. Regrettably, the classical presentations of intussusception such as an abdominal mass, vomiting and bloody stools are not commonly encountered in preterm neonates and in the majority of the cases, the definitive diagnosis is confirmed during the exploration. Additionally, the aforementioned signs along with other signs like distended abdomen and increased gastric residues are common symptoms in both pathologies. Moreover, if diagnosis is mistaken for NEC, the treatment of which, at least initially, is conservative, will lead to a further delay of the diagnosis [29, 30, 50]. Inspite of that delay, this premature group showed a lower overall mortality rate (15%), as opposed to the uniformly fatal outcome in children when left untreated for 2-5 days [51]. Infants suffering from intussusception usually have symptoms that are uniquely limited to the abdomen whilst the overall state does not worsen unless there is bowel perforation, compared to NEC in which abdominal manifestations go ahead in tandem with the deterioration of the general condition [46].

There are no specific radiological findings of IPN. Signs of ileus, such as dilatation of bowel loops and occasionally air-fluid levels, are the most prevalent X-ray findings, whereas X-rays of patients with NEC typically show pneumatosis intestinalis or portal venous gas [9]. Contrast enemas can be diagnostic and therapeutic in older infants; nevertheless, they are less effective effective in preterms because the pathology is mainly restricted to the small bowel in this group, whereas it is usually ileo-colic in older infants [44]. In older infants, abdominal ultrasonography carries a sensitivity of over 98% and specificity of 100% in diagnosing intussusception [52]. However, in neonatal intussusception in general, there are several factors which can alter the sensitivity of ultrasound in diagnosis. First of all, the sigmoid colon lies superficially on the right side in a considerable number of neonates. Together with the presence of marked abdominal gases, the intussusception and the caecal shadow are obscured. Also, the absence of colonic involvement in preterms is definitely an additional factor [34, 52].

When compared to intussusception in infants and full-term neonates in which the ileo-colic type predominates [14], ileo-ileal intussusception is the most prevalent site in preterms with other locations almost non-existent [28]. Beside the location, another source of difference is an identifiable lead point. Unlike intussusception in full term infants where there is sometimes an identifiable lead point [30], the etiology of neonatal intussusception in premature infants is still unknown. Common prenatal injuries that cause intestinal hypoxia/hypoperfusion, dysmotility, and strictures have been proposed as a possible lead point [27].

Unlike intussusception in full-term babies, bowel resection in preterms was commonly indicated. A primary anastomosis was performed in around 46% of IPN cases. Interestingly, there was no statistically significant difference in death rate between those who underwent a primary anastomosis and those who underwent a stoma and mucous fistula, or those who underwent a primary bowel anastomosis in the setting of a perforated gut. To recap, primary anastomosis could be safely done with no increasing morbidity or mortality; hence, obviating the requirment for a second surgery [27]..

This systematic review included 43 studies with a total of 52 cases of IPN reported between 1963 and 2020. Strengths of this report include a thorough search of database, explicit a strict selection criteria for the relevant studies. Furthermore, it summarized a relatively large number of cases of this rare disease, outlined some key features of IPN and how to differentiate it from NEC. However, the main limitation in our study is that it includes only case reports and case series. It is well recognized that case reports remain the lowest rank in the hierarchy of evidence, and though case reports are valuable scoping tools in discovering rare disorders, they have limited strength in the establishment of clear guidelines to differentiate between NEC and IPN. Secondly, the number of cases in our study may not be an accurate one as there are numerous unreported as well as misdiagnosed cases. The latter ones may have been spontaneously reduced or died with their diagnosis being definitely unmasked.

## Conclusion

IPN is a very rare condition that can be deceiving; thus, necessitating a high index of suspicion in order to avoid confusion with the other causes of neonatal bowel obstruction. From all of those causes, it's critical to discriminate it from NEC, that may be addressed conservatively.in the vast majority of cases

## Data Availability

The datasets used and/or analysed during the current study are available from the corresponding author on reasonable request.

## Abbreviations

IPN: Intussusception in preterm neonates
NEC: necrotizing enterocolitis
US: ultrasound

## References

[1] Mannai H, Chourou H, Ksibi I (2017). Acute intussusception in new born: A rare cause of intestinal obstruction. J Gastroenterol Dig Dis.

[2] Kliegman RM, Fanaroff AA (1984). Necrotizing enterocolitis. N Engl J Med.

[3] Columbani PM, Scholz S, Coran AG, Caldamone A, Adzick NS, Krummel TM, Laberge JM, Shamberger R (2012). Intussusception. Pediatric Surgery.

[4] Sterne JA, Hernán MA, Reeves BC, et al. ROBINS-I: a tool for assessing risk of bias in non-randomised studies of interventions. BMJ 201;355:i4919. 10.1136/bmj.i4919 PMC5062054 27733354

[5] Pierson DJ (2009). How to read a case report (or teaching case of the month). Respir Care.

[6] Spencer R (1963). Gastrointestinal hemorrhage in infancy and childhood: 476 cases. Surgery.

[7] Yoo RP, Touloukian RJ (1974). Intussusception in the newborn: A unique clinical entity. J Pediatr Surg.

[8] Stine MJ, Harris H (1982). Intussusception in a premature infant simulating neonatal necrotizing enterocolitis. Am J Dis Child.

[9] Smith VS, Giacoia GP (1984). Intussusception associated with necrotizing enterocolitis. Clin Pediatr.

[10] Glick B, Alpan G, Vinograd I (1985). Meconium plugs and intussusception in a premature infant. Am J Perinatol.

[11] Carman J, Grunebaum M, Gorenstein A (1987). Intussusception in a premature infant simulating necrotising enterocolitis. Z Kinderchir.

[12] Blair GK, Lee JT, Dimmick JE (1990). Postoperative intussusception in a premature infant. J Pediatr Surg.

[13] Rausin L, Khamis J, Paquot JP (1992). Obstruction of unusual origin in a small preterm baby-girl. J Belge Radiol.

[14] Farstad T, Bjordal R, Stake G (1993). Early intussusception in premature infants. Eur J Pediatr.

[15] Price KJ, Roberton NR, Pearse RG (1993). Intussusception in preterm infants. Arch Dis Child.

[16] Iuchtman M, Iurman S, Levin M (1995). Neonatal intussusception misdiagnosed as necrotizing enterocolitis. Am J Perinatol.

[17] Puvabanditsin S, Garrow E, Samransamraujkit R (1996). Postnatal intussusception in a premature infant, causing jejunal atresia. J Pediatr Surg.

[18] Mooney DP, Steinthorsson G, Shorter NA (1996). Perinatal intussusception in premature infants. J Pediatr Surg.

[19] Réguerre Y, de Dreuzy O, Boithias C (1997). An unknown etiology of fetal ascites: Acute intestinal intussusception. Arch Pediatr.

[20] Wang NL, Yeh ML, Chang PY (1998). Prenatal and neonatal intussusception. Pediatr Surg Int.

[21] Stanley P, Aneja R, Isaacson L (1999). Radiology Casebook: Intussusception in a Premature Infant: A Case Report. J Perinatol.

[22] Gorgen-Pauly U, Schultz C, Kohl M (1999). Intussusception in preterm infants: Case report and literature review. Eur J Pediatr.

[23] Hirokawa S, Uotani H, Yoshida T (2001). Ileoileal intussusception and ileal stricture associated with necrotizing enterocolitis in a premature infant: Report of a case. Surg Today.

[24] Goo HW, Kim EA, Pi SY (2002). Sonographic diagnosis of neonatal intussusception with perforation in a premature neonate. Am J Roentgenol.

[25] Margenthaler JA, Vogler C, Guerra OM (2002). Pediatric surgical images: Small bowel intussusception in a preterm infant. J Pediatr Surg.

[26] Nock ML, Wilson-Costello D (2002). Intussusception in a premature neonate. Clin Pediatr..

[27] Avansino JR, Bjerke S, Hendrickson M (2003). Clinical features and treatment outcome of intussusception in premature neonates. J Pediatr Surg.

[28] Martínez Biarge M, García-Alix A, Luisa del Hoyo M (2004). Intussusception in a preterm neonate; A very rare, major intestinal problem - systematic review of cases. J Perinat Med.

[29] Ueki I, Nakashima E, Kumagai M (2004). Intussusception in neonates: analysis of 14 Japanese patients. J Paediatr Child Health.

[30] Slam KD, Teitelbaum DH (2007). Multiple sequential intussusceptions causing bowel obstruction in a preterm neonate. J Pediatr Surg.

[31] Al-Jahdali A, Lees GM, Gay DP, Al-Sairafi R (2009). Colocolic intussusception in a preterm infant with intestinal malrotation. J Pediatr Surg.

[32] Boubal M, Jacquot A, Baud C (2010). Acute intussusception, a rare cause of small bowel obstruction in premature neonates: the advantages of early diagnosis. Arch Pediatr.

[33] Kim S, Lee JJ, Yoo BH (2010). Double intussusception in a preterm infant. Korean J Perinatol.

[34] Shad J, Biswas R. Ileo-colic intussusception in premature neonate. BMJ Case Rep. 2011;2011. 10.1136/bcr.11.2011.5109 PMC3233927 22669773

[35] Shima Y, Kumasaka S, Yashiro K (2012). Intussusception in an extremely premature infant following bacterial sepsis. Eur J Pediatr.

[36] Altuntas N, Boyunaga O, Karabulut R (2015). Ileo-ileal intussusception in a premature neonate: an unusual cause of NEC in premature babies. JCPSP.

[37] Kim HS, Kim HA, Kim SH (2014). Multiple intussusceptions in an extremely premature infant. Korean J Perinatol.

[38] Taşkınlar H, Gündoğdu G, Çelik Y (2014). Challenging diagnosis between intussusception and necrotizing enterocolitis in premature infants. Pediatr Int..

[39] Prakash A, Doshi B, Singh S (2015). Intussusception in a premature neonate: A rare and often misdiagnosed clinical entity. Afr J Pediatr Surg.

[40] Park JY, Kim YG, Lee NM (2015). Double Intussusceptions with Necrotizing Enterocolitis Diagnosed in a Premature Infant. Neonatal Med..

[41] Patel R, Tan YW, Patil S (2016). Neonatal ileo-ileal and ileo-cloacal exstrophic synchronous dual intussusceptions in a preterm infant with cloacal exstrophy. World J Nephrol Urol..

[42] Aydin E (2018). Intussusception in a preterm newborn. Pediatr Neonatol.

[43] Tepmalai K, Naowapan T, Singhavejsakul J (2017). Intussusception in premature baby: Unusual cause of bowel obstruction and perforation. J Neonatal Surg.

[44] Diwakar K, Al Awad E, Hasan S (2017). Multiple intussusceptions associated with meconium plugs: a case and literature review. Res Pediatr Neonatol.

[45] Pawar S, Shyamsunder T, Reddy S (2018). A case report, ileo-ileal intussusception in preterm neonate: An unusual location. Acad J Pediatr Neonatol.

[46] Raza HA, Basamad MS, El Komy MS (2014). Diagnosing intussusception in preterm neonates: case report and overview. J Clin Neonatol.

[47] Kotb M, Abdelatty M, Fawzy O (2019). Intussusception in preterm neonate. J Pediatr Surg..

[48] Hukeri AR, Gupta A, Kothari P (2019). Bowel intussusception in premature baby: Needs high degree suspicion for early detection. J Clin Neonatol..

[49] Rachelson MH, Jernigan JP, Jackson WF (1955). Intussusception in the newborn infant: With spontaneous expulsion of the intussusceptum; A case report and review of the literature. J Pediatr..

[50] Al Salem AH, Hasbash BM (2000). Ileo-ileal intussusception: a report of 4 cases. Ann Saudi Med.

[51] Chalya PL, Kayange NM, Chandika AB (2014). Childhood intussusceptions at a tertiary care hospital in northwestern Tanzania: a diagnostic and therapeutic challenge in resource-limited setting. Ital J Pediatr..

[52] Williams H (2008). Imaging and intussusception. Arch Dis Child Educ Pract.

